# Responses of *Sphagneticola trilobata*, *Sphagneticola calendulacea* and Their Hybrid to Drought Stress

**DOI:** 10.3390/ijms222011288

**Published:** 2021-10-19

**Authors:** Qilei Zhang, Jundong Huang, Weiqian Ke, Minling Cai, Guangxin Chen, Changlian Peng

**Affiliations:** 1 Guangzhou Key Laboratory of Subtropical Biodiversity and Biomonitoring, Guangdong Provincial Key Laboratory of Biotechnology for Plant Development, School of Life Sciences, South China Normal University, Guangzhou 510631, China; dalei45666@163.com (Q.Z.); dajun45666@126.com (J.H.); keweiqscnu@163.com (W.K.); cai2214@126.com (M.C.); gxchen2019@126.com (G.C.); 2 Research Institute of Tropical Forestry, Chinese Academy of Forestry, Guangzhou 510520, China

**Keywords:** abscisic acid, antioxidant, biological invasion, drought, hybrid, *Sphagneticola* *trilobata*

## Abstract

*Sphagneticola trilobata* is an invasive plant in South China. A hybrid between *S*. *trilobata* and *Sphagneticola calendulacea* (a native related species) has also been found in South China. The drought resistance of *S*. *calendulacea*, *S*. *trilobata* and their hybrid was studied in this paper. Under drought stress, the leaves of *S*. *trilobata* synthesized more abscisic acid (ABA) than those of the other species to reduce stomatal opening and water loss. The activities of antioxidant enzymes were the highest in *S*. *trilobata* and the lowest in *S. calendulacea*. The leaves of *S. calendulacea* suffered the most serious damage, and their maximum photochemical efficiency was the lowest. RNA-sequencing ware used to analyze the expression levels of genes in ABA, antioxidant enzyme, sugar and proline synthesis and photosynthesis pathways. Further real-time PCR detection verified the RNA-sequence results, and the results were in accordance with the physiological data. The results showed that *S*. *trilobata* was the most drought tolerant, and the drought tolerance of the hybrid did not show heterosis but was higher than *S. calendulacea*. Therefore, compared with *S. trilobata* and the hybrid, the population number and distribution of *S. calendulacea* may be less in arid areas.

## 1. Introduction

Under the influence of global climate change, extremely harsh environments are becoming more frequent and serious, including arid environments. Some studies have shown that arid, semi-arid and dry sub humid areas account for about 41% of the global land area, and the arid areas will gradually increase with global warming [1]. Soil moisture is one of the most important components of soil, and it is also the basic condition for the survival of terrestrial plants. The distribution pattern of water determines the spatial pattern of vegetation. In arid areas, it is the main environmental factor restricting plant growth. Soil moisture also affects the richness, species and distribution of vegetation [2]. Previous studies showed that when the temperature was stable, the vegetation structure was easily affected by water. The change in rainfall will lead to a change of vegetation in the area by about 60%, and the composition of vegetation will also change. In arid areas, plant coverage and species diversity decreased, and the proportion of deep root plants and drought tolerant plants increased [3,4].

Drought stress is one of the most important abiotic stresses limiting plant growth. Drought stress is mainly characterized by the destruction of the osmotic balance in cells, which leads to a series of physiological, biochemical and molecular changes in plants and seriously affects their growth and development [5]. In the process of drought stress, to reduce the loss of water, plants reduce the opening of their stomata and even close them [6]. Drought stress is often accompanied by oxidative stress, resulting in the production and accumulation of a large number of reactive oxygen species (ROS) in cells. This process results in lipid peroxidation and protein oxidation, which can reduce the chlorophyll content and affect protease activity, thus affecting photosynthesis and reducing plant biomass [7].

In the long-term evolution of plants, to reduce the damage caused by drought stress, a series of coping strategies have evolved. The expression of genes related to abscisic acid (ABA) synthesis was significantly upregulated in plants, which increased ABA synthesis and transduction, decreased the stomatal opening, and reduced water loss due to transpiration [8]. To reduce water loss, plants will increase their osmotic adjustment substances and reduce the water potential. The proline and soluble sugar contents in plant cells will increase significantly. Furthermore, the antioxidant capacity of plant cells will improve. To remove excessive ROS in cells, plants will increase the content of enzymatic antioxidant substances and nonenzymatic antioxidant substances in their cells. In a study on model plants such as maize (*Zea mays*), rice (*Oryza sativa*), tobacco (*Nicotiana tabacum*) and *Arabidopsis thaliana*, it was found that drought stress can reduce crop yield and increase the production of ROS in plant cells, leading to an increase in cell membrane lipid peroxidation and cell permeability. At the same time, the content of antioxidant substances and the activity of antioxidant enzymes in cells will also be significantly increased [9,10,11,12].

Under drought stress, not only physiological metabolism but also molecular expression patterns will change. RNA sequencing (RNA-seq) is a common technique and powerful method for transcriptional analysis, and transcripts have been analyzed during plant responses to drought stress. Min et al. [13] used RNA-seq to analyze the response of maize seedlings to drought stress at three time points. Opitz et al. [14] used transcriptional groups to study the difference in gene expression in the root system of maize under drought conditions for six and 24 h. Zhang et al. [15] analyzed the gene expression patterns of maize seedlings during drought treatment and the water recovery period using RNA-seq data. In invasive plants, RNA-seq was used to analyze the invasion mechanism of *Solidago Canadensis* [16,17].

*Sphagneticola trilobata* (L.) Pruski, a perennial herb of the Compositae that originated in South and Central America, is an invasive plant in South China. A hybrid between *S*. *trilobata* and its local relative, *S*. *calendulacea*, was found in South China [18]. Previous studies have shown that the response of hybrids to nitrogen deposition and their tolerance to low temperature and low light stress did not show heterosis compared with their parents [19,20]. Our recent studies showed that the tolerance of hybrids to cadmium stress showed heterosis, and their adaptability was higher than that of native and invasive species [21]. However, it is still unknown whether these hybrids have a superior parental advantage in tolerance to drought stress. In dry regions, water is the main environmental factor that restricts the growth of plants. Soil water affects the richness, species and distribution of vegetation [2]. In this paper, *S*. *calendulacea*, *S*. *trilobata* and their hybrid were used as research materials to carry out drought stress experiments and to compare the changes in physiological indexes and gene expression levels of the three species in order to explore the differences in the responses of the three species to drought stress and to predict the changes in the distribution of the three species in dry regions in the future.

## 2. Results

### 2.1. Phenotypic Characteristics and Water and ABA Contents

**Figure 1:**
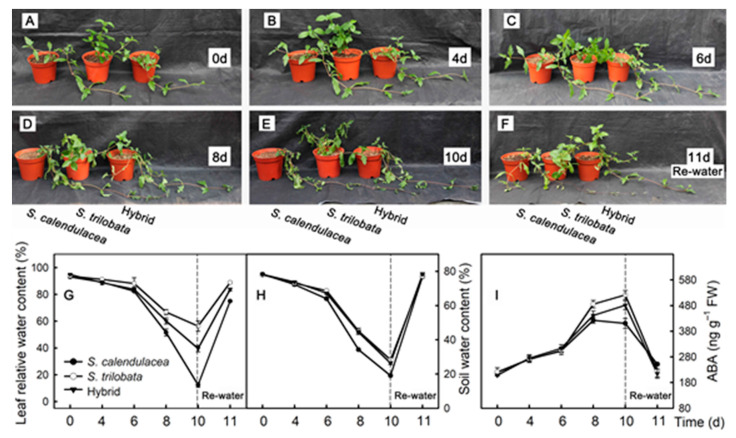
Phenotype and moisture content. Changes of phenotype (**A**–**F**), leaf relative water content (**G**), soil water content (**H**) and ABA content (**I**) of *S*. *calendulacea*, *S*. *trilobata* and their hybrid under drought stress and re-watered.

As the time under drought stress increased, the leaves of both parents and hybrids wilted. The leaves of *S. calendulacea* wilted first. After eight days of drought, the leaves of both *S. calendulacea* and the hybrid wilted noticeably (Figure 1D). After 10 d of drought, the leaves of both parents and the hybrid wilted due to water loss (Figure 1E). After 10 d of drought, the two parents and hybrids were rewatered for one d. The results showed that *S. trilobata* and the hybrid could recover completely, while *S. calendulacea* could not (Figure 1F).

After drought treatment, the relative leaf water content and soil water content of both parents and hybrids decreased gradually (Figure 1G,H). The relative water content of *S. calendulacea* leaves decreased the fastest, the *S. trilobata* leaves decreased the slowest, and the hybrids were in the middle. The rate of decline of the soil water content was the fastest in *S. calendulacea* and was slower in *S. trilobata* and the hybrid. After rewatering, the soil water content of the two parents and hybrid recovered to the original level, but the recovery level of the leaf’s relative water content was different. The leaf’s relative water content of *S. calendulacea* was lower than that of *S. trilobata* and the hybrid. As the time under drought stress increased, the ABA content in the leaves of the two parents and hybrid increased gradually (Figure 1I). At eight d, the ABA content in the leaves of *S. trilobata* was higher than that in the leaves of *S. calendulacea* and the hybrid. After rewatering, the ABA content in the leaves of the two parents and hybrid decreased.

### 2.2. Stomatal Size, Proline and Soluble Sugar Content

**Figure 2:**
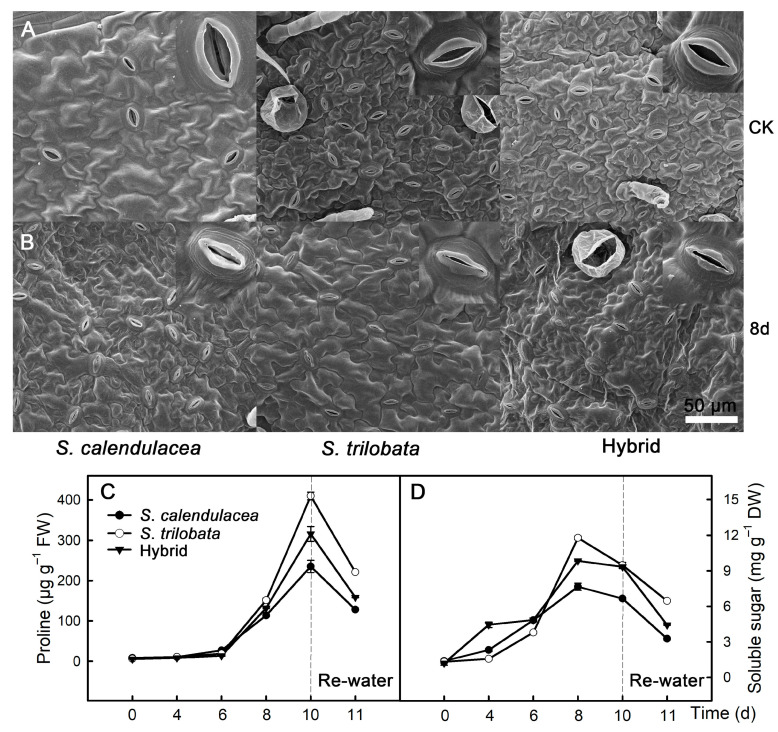
Pore size and osmoregulation substance content after eight days of normal irrigation (CK) and drought stress treatment (8 d), leaf stomatal size (**A**,**B**), and changes of proline (**C**) and soluble sugar (**D**) contents in leaves of *S*. *calendulacea*, *S*. *trilobata* and their hybrid under drought stress and re-watered.

The stomatal opening under drought treatment for eight d was smaller than that under normal irrigation (CK) (Figure 2A,B). With the extension of drought time, the proline content in the leaves of the two parents and hybrid increased gradually. The proline content in the leaves of *S. trilobata* was higher than that of the hybrid after 10 d of drought, and the proline content in *S. calendulacea* leaves was the lowest. After rewatering, the proline content in the leaves of both parents and the hybrid decreased (Figure 2C). The soluble sugar content in the leaves also increased as the time under drought stress increased; it reached its highest value at eight d of drought and decreased at 10 d of drought (Figure 2D). At eight d of drought, the soluble sugar content in the leaves of *S. trilobata* was higher than that of those of the hybrid, and it was lowest in *S. calendulacea*. After rewatering, the soluble sugar content in the leaves of both parents and the hybrid decreased.

### 2.3. Malondialdehyde Content and Antioxidant Capacity

**Figure 3:**
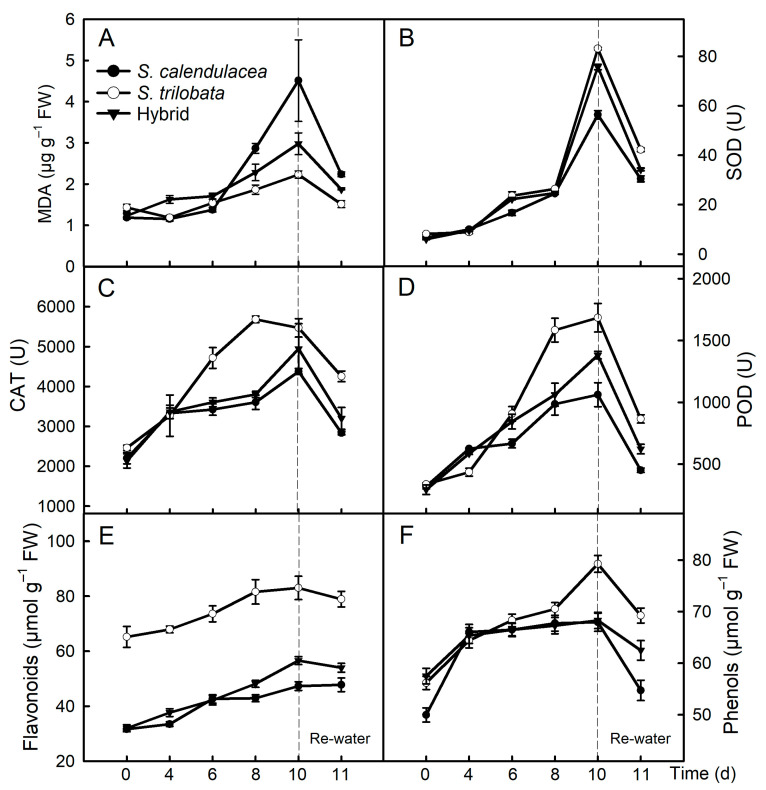
Antioxidant enzyme activity and antioxidant substance content. Changes of the activities of superoxide dismutase (SOD, **B**), catalase (CAT, **C**), peroxidase (POD, **D**) and total antioxidant capacity (TAC, **D**), and the contents of malondialdehyde (MDA, **A**), flavonoids and phenols (**E**,**F**) in leaves of *S*. *calendulacea*, *S*. *trilobata* and their hybrid under drought stress and re-watered.

During the drought treatment, the malondialdehyde (MDA) content in the leaves of both parents and the hybrid increased gradually (Figure 3A). The MDA content in the leaves of *S. calendulacea* was higher than that of those of *S. trilobata* and the hybrid, and it was the lowest in the leaves of *S. trilobata*. After rewatering, the MDA content in the leaves decreased.

As the time under drought stress increased, the SOD, CAT and POD activities in the leaves of the two parents and the hybrid gradually increased (Figure 3B–D). Among them, the enzyme activity in *S. trilobata* leaves was the highest and that in *S. calendulacea* leaves was the lowest. After rewatering, the SOD, CAT and POD activities in the leaves of both parents and the hybrid decreased. Flavonoids and total phenols, as nonenzymatic antioxidants in plants, can scavenge ROS and reduce oxidative stress. During drought, the accumulation of flavonoids and total phenols in the leaves of both parents and the hybrid increased gradually (Figure 3E,F). The contents of flavonoids and total phenols in the leaves of *S. trilobata* were higher than that in the leaves of *S. calendulacea* and the hybrid. After rewatering, the flavonoid and total phenol contents in the leaves of both parents and the hybrid decreased.

### 2.4. Chlorophyll Fluorescence Parameters and Photosynthetic Capacity

**Figure 4:**
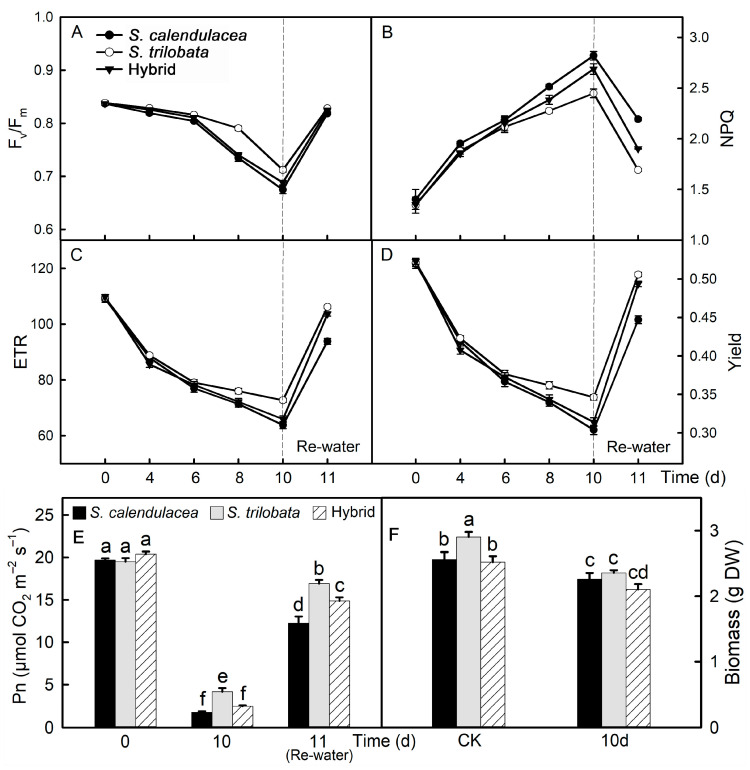
Chlorophyll fluorescence parameters and photosynthetic capacity. Changes of the maximum photochemical efficiency (F_v_/F_m_, **A**), thermal dissipation (NPQ, **B**), electron transport rate (ETR, **C**), actual photochemical efficiency (yield, **D**) and net photosynthetic rate (Pn, **E**) in leaves of *S*. *calendulacea*, *S*. *trilobata* and their hybrid under drought stress and re-watered. Comparison of biomass (**F**) between control (CK) and drought for 10 days (10 d). Different lowercase letters (a, b, c, d, e, f) above bars indicate statistical significance (*p* < 0.05).

During drought, F_v_/F_m_, ETR and Yield of the leaves of both parents and the hybrid gradually decreased (Figure 4A–D). After six d of drought, the F_v_/F_m_ in the leaves of *S. calendulacea* and the hybrid decreased faster, and this value was the highest in the leaves of *S. trilobata*. ETR and yield were higher in the leaves of *S. trilobata* than in those of the hybrid and *S. calendulacea* after six d of drought. After rewatering, the F_v_/F_m_, ETR and yield in the leaves of both parents and the hybrid increased. The value of thermal dissipation (NPQ) increased gradually in the leaves of both parents and the hybrid during drought (Figure 4B). After sixd of drought, the NPQ in the leaves of *S. calendulacea* was the highest, while it was the lowest in the leaves of *S. trilobata*.

As the time under drought stress increased, the Pn in the leaves of the two parents and hybrid decreased gradually (Figure 4E). This value was higher in the leaves of *S. trilobata* than that of *S. calendulacea* and the hybrid. After rewatering, the Pn in the leaves of both parents and the hybrid increased. Drought stress also affected the growth of the two parents and hybrid. After 10 days, compared with the control group (CK), the biomass of *S. trilobata*, *S. calendulacea* and the hybrid decreased by 12%, 19% and 17%, respectively, under drought stress (10 d) (Figure 4F).

### 2.5. Number of Differentially Expressed Genes

There were 547 differentially expressed genes (DEGs) in *S. calendulacea* leaves, 307 of which were upregulated and 240 of which were downregulated. There were 1932 DEGs in *S. trilobata* leaves, 781 of which were upregulated and 1151 were downregulated. There were 1115 DEGs in hybrid leaves, 822 of which were upregulated and 293 were downregulated (Table 1). The KEGG database was used to classify and enrich the DEGs (Supplementary Figures S3–S8).

### 2.6. Expression of Genes Related to ABA, Sugar and Proline Synthesis

**Figure 5:**
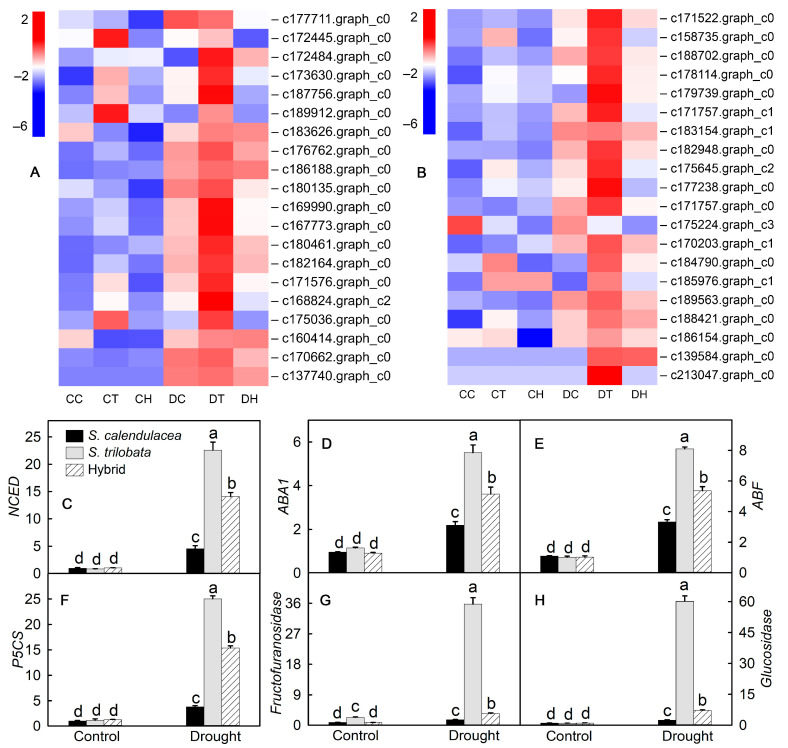
Heatmap of gene expression and results of real-time PCR validation. Heatmap of genes related to abscisic acid (**A**), sugar and proline (**B**) synthesis and the results of real-time PCR validation (**C**–**H**) under normal irrigation (control) and drought treatment (drought) in leaves of *S*. *calendulacea*, *S*. *trilobata* and their hybrid. Different lowercase letters (a, b, c, d) above bars indicate statistical significance (*p* < 0.05). CC: control of *S. calendulacea*; CT: control of *S.* *trilobata*; CH: control of the hybrids; DC: drought stress treatment of *S. calendulacea*; DT: drought stress treatment of *S.* *trilobata*; DH: drought stress treatment of the hybrids.

ABA is an endogenous hormone in plants. An increase in ABA content can effectively reduce stomatal opening, and the ABA content in plant leaves will increase significantly under drought conditions. Figure 5A presents a heatmap of DEGs in ABA synthesis and transduction pathways. Most genes were upregulated in both the parents and hybrid after drought treatment. *NCED* and *ABA1* are the key enzymatic genes in the ABA synthesis pathway, and their expression levels are positively correlated with ABA content; *ABF* is a key enzymatic gene that is positively regulated in the process of ABA signal transduction. The results of real-time PCR showed that the relative expression levels of the *NCED*, *ABA1* and *ABF* genes were significantly higher than that of the control group after drought treatment. After drought treatment, the relative expression levels of *NCED*, *ABA1* and *ABF* genes in the leaves of *S. trilobata* were the highest and were significantly higher than those of *S. calendulacea* and the hybrid; the expression levels were the lowest in the leaves of *S. calendulacea* (Figure 5C–E). Sugar and proline can regulate the osmotic pressure of plant cells. In an environment facing a water shortage, the increase in the sugar and proline content can reduce the water potential of cells. The results showed that the expression of genes in the sugar and proline synthesis pathway was upregulated after drought treatment (Figure 5B). *P5CS* is an enzymatic gene involved in proline synthesis, and its expression can promote proline synthesis. The *fructofuranosidase* and *glucosidase* genes encode enzymes in the sugar synthesis pathway. Their expression can increase the content of soluble sugar. The expression of *P5CS*, *fructofuranosidase* and *glucosidase* significantly increased after drought, and it was the highest in *S. trilobata* leaves and the lowest in *S. calendulacea* leaves (Figure 5F–H).

### 2.7. Expression of Genes Related to Antioxidant Enzyme Synthesis

**Figure 6:**
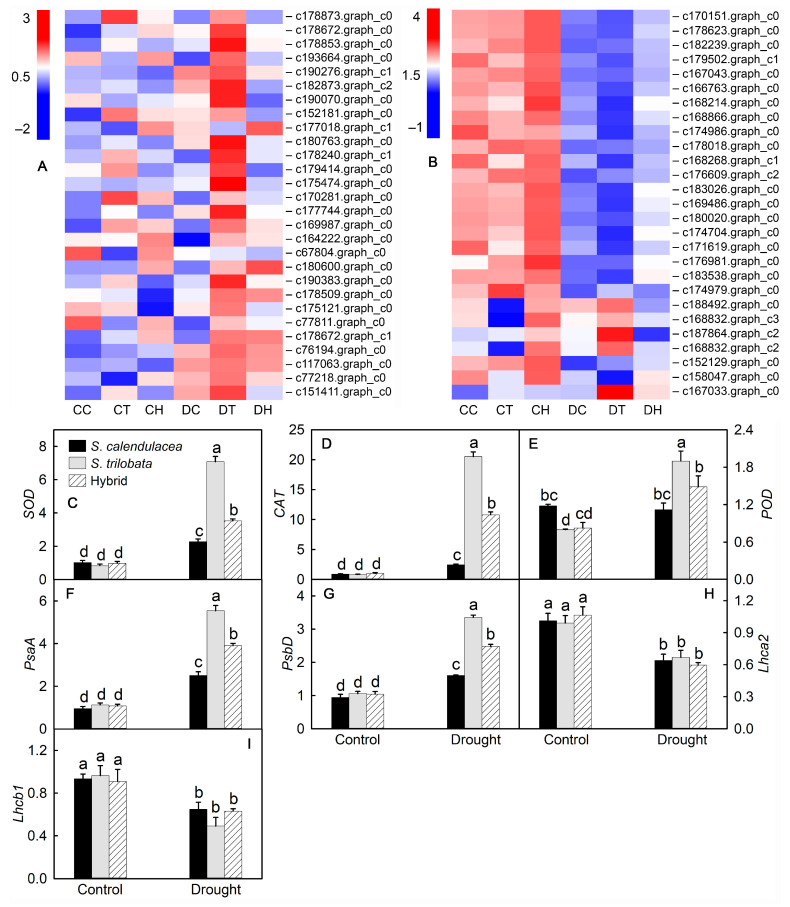
Heatmap of gene expression and results of real-time PCR validation. Heatmap of genes related to antioxidant enzymes synthesis (**A**) and photosynthetic pathway (**B**), and the results of real-time PCR validation (**C**–**I**) under normal irrigation (control) and drought treatment (drought) in leaves of *S*. *calendulacea*, *S*. *trilobata* and their hybrid. Different lowercase letters (a, b, c, d) above bars indicate statistical significance (*p* < 0.05). CC: control of *S. calendulacea*; CT: control of *S.* *trilobata*; CH: control of the hybrids; DC: drought stress treatment of *S. calendulacea*; DT: drought stress treatment of *S.* *trilobata*; DH: drought stress treatment of the hybrids.

The antioxidant enzymes SOD, CAT and POD can scavenge ROS and reduce oxidative stress in plant cells. *SOD*, *CAT* and *POD* are the main regulatory genes in the synthesis of antioxidant enzymes. The results showed that the expression of *SOD*, *CAT* and *POD* was upregulated after drought treatment (Figure 6A). The results of real-time PCR showed that the expression of antioxidant enzyme genes in the leaves of the two parents and hybrid increased significantly under drought stress (Figure 6C–D). The relative expression of antioxidant enzymatic genes in *S. trilobata* was the highest, which was significantly higher than that of *S. calendulacea* and the hybrid, and it was the lowest in *S. calendulacea*.

### 2.8. Expression of Genes Related to Enzyme Synthesis in Photosynthesis

Photosynthesis is the basis for the synthesis and accumulation of organic matter in higher plants and involves the absorption of light energy and electron transfer by leaves. Drought not only affects the water content of plant leaves but also affects the electron transfer of PSI and PSII in leaves. The heatmap of photosynthesis pathway-related gene expression in the drought treatment group and control group was shown in Figure 6B. Some genes were upregulated and some were downregulated after drought stress. The downregulated genes were mainly involved in the synthesis of PSI and PSII antenna proteins, while the upregulated genes were mainly involved in the synthesis of the PSI and PSII electron transport chain protein complex. *PsaA* and *PsbD* are involved in the synthesis of protein complexes in the electron transport chain of PSI and PSII, respectively; *Lhca2* and *Lhcb1* are involved in the synthesis of PSI and PSII antenna proteins, respectively. Their expression was positively correlated with the electron transfer rate and light energy capture ability in photosynthesis. The results of real-time PCR showed that the relative expression levels of the *PsaA* and *PsbD* genes were significantly upregulated after drought treatment (Figure 6F,G), while those of the *Lhca2* and *Lhcb1* genes were significantly downregulated (Figure 6H,I). The relative expression levels of the *PsaA* and *PsbD* genes were the highest in the leaves of *S. trilobata*, which were significantly higher than those of *S. calendulacea* and the hybrid, and the lowest expression was in the leaves of *S. calendulacea*. There was no significant difference in the relative expression of the *Lhca2* and *Lhcb1* genes between the two parents and the hybrid.

## 3. Discussion

It can be seen from the phenotypic changes of the plant that the leaves of both parents and the hybrid wilted under drought stress (Figure 1). *S. calendulacea* leaves wilted after eight d of drought, and the phenotype of *S. calendulacea* could not completely recover after being rewatered after 10 days of drought, indicating that *S. calendulacea* was under the most serious stress during drought treatment. In addition, the relative water content in the leaves of *S. calendulacea* decreased fastest, while that in the leaves of *S. trilobata* decreased the slowest, and the rate in the hybrid was between that of the two parents (Figure 1G). Stomatal openings will decrease in plant leaves under drought stress. The results of studies in *Arabidopsis* and rice showed that the stomatal opening of leaves can be significantly reduced under drought stress, thus maintaining the moisture content in leaves and soil; plants with more sensitive stomatal regulation are more drought resistant [8,11,22]. Our results showed that the stomatal opening was smaller under drought stress than under normal conditions (Figure 2A,B), and the stomatal opening of *S. trilobata* was smaller than that of the hybrid and *S. calendulacea*. Stomatal movement is mainly caused by the change in osmotic pressure inside and outside the guard cell, and potassium plays an important role in regulating the osmotic pressure inside and outside the guard cell [23]. The results showed that the guard cells of plants in the control group accumulated a large amount of potassium ions, and the stomatal opening was relatively large. After drought treatment, potassium ions in the guard cells of leaves decreased significantly, and the stomatal opening was relatively small (Supplementary Figure S1). Previous studies also found that potassium ion flow on the membrane of guard cells can improve the opening of stomata, and a large amount of potassium ions can be detected in open guard cells, while the potassium content in guard cells in the closed state is lower [24,25].

ABA is one of the hormones closely related to stomatal movement under drought stress. This study found that under drought stress, the expression of genes related to ABA synthesis and ABA content in the leaves of the two parents and the hybrid gradually increased (Figure 5A). After 8 d of drought treatment, the ABA content in the leaves of *S. trilobata* was the highest. This result indicated that *S. trilobata* could rapidly synthesize more ABA in response to drought stress. Additionally, in response to drought stress, endogenous ABA levels increase rapidly, which in turn activates specific signaling pathways and changes gene expression [26,27]. The heatmap showed that most of the genes related to ABA synthesis and transduction were upregulated under drought stress. Among them, *ABA1* and *NCED* are the key enzymatic genes of the ABA biosynthesis pathway in higher plants [28,29]. The results showed that the relative expression of the *NCED* gene in the leaves of *S. calendulacea*, *S. trilobata* and their hybrid increased by 3.7, 26.7 and 12.8 times after drought treatment, respectively. The relative expression of the *ABA1* gene was upregulated by 1.3, 3.8 and 2.9 times in the leaves of *S. calendulacea*, *S. trilobata* and their hybrid, respectively (Figure 5C,D). In addition, we detected that the expression of *ABF* was significantly upregulated after drought stress, and *ABF* played a positive regulatory role in ABA signal transduction under drought stress [30]. These results suggested that ABA-mediated signal transduction was involved in the response of the three species to drought and that ABA was more sensitive in the regulation of *S. trilobata* leaves.

Under drought stress, a large number of ROS, including superoxide anion and hydrogen peroxide, accumulate in plants, which results in oxidative stress [10]. To remove excessive ROS in cells, plants synthesize a large number of antioxidants, including antioxidant enzymes and nonenzymatic substances. The results showed that the content of flavonoids and total phenols and the activity of antioxidant enzymes (SOD, CAT, POD) in the leaves of the three species increased gradually under drought stress (Figure 3). This may be due to the increase in the accumulation of ROS in cells under drought stress, which leads to an increase in antioxidant enzyme activity in plant cells [31]. In the late drought stress period, SOD, CAT and POD activities in the leaves of *S. trilobata* were higher than that in the leaves of *S. calendulacea* and the hybrid. Based on the RNA-seq results, the expression of these antioxidant enzyme genes significantly increased under drought conditions, among which the relative expression of *SOD*, *POD* and *CAT* genes was the highest in the leaves of *S. trilobata* (Figure 6). This indicated that the SOD, CAT and POD activities were higher in *S. trilobata* leaves under drought stress. Under drought stress, the accumulation of ROS and MDA in the leaves of the three species increased (Figure 3A; Supplementary Figure S2). These compounds were the least abundant in *S. trilobata*, the most abundant in *S. calendulacea* and in between the two levels in the hybrid, which may be related to the antioxidant capacity of the three species.

Photosynthesis is the basic physiological process of plant growth and development, but drought can significantly affect this process [32]. Photosynthesis includes light reactions and carbon fixation. In the light reaction stage, drought affects the enzyme activities in PSI and PSII, thus affecting light energy absorption and electron transfer. The RNA-seq data showed that under drought stress, the expression of protein-coding genes related to light energy absorption was downregulated, while the expression of protein-coding genes related to electron transport was upregulated, suggesting that the three species reduced their ability to absorb light energy and increased their ability to transport electrons under drought stress. This may be because excess electron flow will produce more ROS and cause damage to cells [33]. Under drought stress, the relative expression of the *PsaA* and *PsbD* genes was significantly upregulated, and it was highest in *S. trilobata* leaves, while the relative expression of the *Lhca2* and *Lhcb1* genes was significantly downregulated (Figure 6F–I).

## 4. Materials and Methods

### 4.1. Plant Materials

After the leaves were removed, the stem was cut into stem segments containing two stem nodes each. The stem segments were put into an opaque open glass bottle for rooting and germination in an incubator under a light intensity of 100 μmol m^−2^ s^−1^, a photoperiod of 14 h (dark for 10 h), and a temperature of 25 °C. After two weeks of cultivation, the seedlings were planted in garden nutrient soil (Jiffy, The Netherlands, http://www. jiffygroup.com/jiffy_product_category/substrates/; accessed on 13 November 2019). They were then placed in the experimental garden and protected from the rain with transparent polymethyl methacrylate. When a plant grew five pairs of leaves, it was no longer watered in order to initiate drought treatment. During the experiment, there were five biological repetitions and three independent experiments.

### 4.2. Determination of Soil and Leaf Water Contents and Stomatal Observations

After the soil was removed, the weight was recorded as the total soil mass, and after drying, the weight of the soil was recorded as the dried soil mass. The soil moisture content (%) = (total soil mass − dried soil mass)/total soil mass × 100%. The weight of fresh leaves after being reduced was recorded as *A*1. The leaves were then completely immersed in water until the weight of the leaves no longer increased, and that weight was recorded as *A*2. Then the leaves were exposed to 105 °C for 20 min and dried at 75 °C to a constant weight, and this weight was recorded as *A*3. Relative water content (%) = (*A*1 − *A*3)/(*A*2 − *A*3) × 100%.

Stomatal observations were performed according to Zhang et al. [34] (2019a). The blade was cut into 2 × 2-mm fragments, and the fragments were then placed in a fixing solution containing 2.5% glutaraldehyde and 2% polyoxymethylene and treated at 4 °C for more than 12 h in the dark. The leaves were t hen dehydrated with different concentrations of ethanol (30%, 50%, 70%, 80%, 90% and 100%). The ethanol solution was changed every 20 min. The dehydrated leaves were dried by the CO_2_ critical point drying method, and then a 30 nm gold layer was sprayed on the surface of the leaves. The stomatal size was recorded by scanning electron microscopy (Q25, FEI, Hillsboro, OR, USA).

### 4.3. Determination of the ABA, Proline and Soluble Sugar Contents

The content of ABA was determined using an ELISA kit (Zike Shenzhen). First, 0.15 g of leaves was added into a precooled mortar; then, 0.8 mL of phosphate buffer (pH 7.4, concentration is 0.05 M) was added, and the sample was ground on ice. The ground homogenate was poured into a 1.5 mL centrifuge tube, and the mortar was moistened with 0.2 mL of phosphate buffer, which was then poured into the same 1.5 mL centrifuge tube and allowed to stand at 4 °C for 2 h. The centrifuge tube containing the tissue homogenate was centrifuged at 5000× *g* for 10 min at 4 °C. The supernatant was collected and then determined according to the manufacturer’s instructions

0.2 g of sample was added to 10 mL of 80% ethanol with 0.01 g of active carbon. The extract was filtered after extraction in the dark for 1 h. Next, 1 g of zeolite was added, and the sample was oscillated for 15 min and then centrifuged at 3000× *g* for 5 min. Then, 2 mL of supernatant, 2 mL of acetic acid and 2 mL of ninhydrin solution (1.25 g of ninhydrin dissolved in 30 mL of acetic acid and 20 mL of 6 m phosphoric acid) were mixed and boiled for 20 min. A UV-2450 spectrophotometer (Shimadzu, Tokyo, Japan) was used to determine the absorbance at 517 nm. Different concentrations of proline were used as standard curves to calculate the concentration.

Determination of soluble sugar was done by anthrone colorimetry. The leaves were washed with deionized water and heated at 105 °C for 20 min and then 70 °C to a constant weight. The dried leaves were ground and passed through a 0.425 mm mesh. 10 mg of the sample was weighed and placed into a 20 mL centrifuge tube. Then, 10 mL of deionized water was added, and the sample was placed in a water bath at 80 °C for 1 h. Next, 40 mg of activated carbon was added to the extraction solution, and then the mixture was reversed and decolorized at 80 °C for 30 min. After filtration, 1 mL of solution was put into a new test tube, and 10 mL of anthrone sulfuric acid was added (first, 76 mL of concentrated sulfuric acid was diluted with 30 mL of deionized water, and then 150 mg of anthrone was dissolved in 100 mL of diluted sulfuric acid). Samples were places in a water bath at 90 °C for 15 min, and then the absorbance was measured at 620 nm with a UV-2450 spectrophotometer (Shimadzu, Tokyo, Japan) after cooling. Different concentrations of glucose were used as standard curves to calculate the soluble sugar content in the sample.

### 4.4. Determination of Enzyme Activity, Antioxidants and MDA

Leaves (0.1 g) were ground with 2 mL of enzyme extract (pH 7.8, 0.05 M phosphate buffer containing 0.1 M EDTA, 0.1% Triton X-100 and 2% PVP) on ice. The sample was centrifuged at 12,000× *g* for 20 min at 4 °C, and then the supernatant was used for enzyme activity determination.

The activity of catalase (CAT) in the supernatant was determined and adjusted appropriately according to the method described by Chance and Maely [35]. First, 0.1 mL of enzyme extract was mixed with 2.9 mL of 30 mM H_2_O_2_ in a quartz cuvette. After 15 s of reaction, the absorbance was recorded at 240 nm nine times (20 s each time) with a UV-2450 spectrophotometer (Shimadzu, Tokyo, Japan). A decrease of 0.01 in absorbance per minute per gram of plant tissue sample was regarded as one cat activity unit.

The activity of peroxidase (POD) in leaves was determined and adjusted appropriately according to the method proposed by Chance and Maely [35]. First, 0.1 mL of enzyme extract was mixed with 50 mM phosphate buffer (1.875 mL, pH 7.0), 30 mM H_2_O_2_ (1 mL) and guaiacol (0.025 mL) in a quartz dish. A UV-2450 spectrophotometer (Shimadzu, Tokyo, Japan) was used to record nine measurements (20 s each time) at 470 nm. An increase of 0.01 in absorbance per minute per gram of plant tissue sample was regarded as one POD activity unit.

The activity of superoxide dismutase (SOD) in leaves was determined and adjusted according to the method of Giannopolitis and Ries [36]. First, 0.1 mL of enzyme extract was mixed with 50 mM phosphate buffer (1.7 mL, pH 7.8), 130 mM methionine (0.3 mL), 0.75 mM tetrazolium blue (0.3 mL), 0.1 mM EDTA-Na_2_ (0.3 mL) and 0.02 mM riboflavin (0.3 mL) in a 5 mL centrifuge tube and irradiated with 4500 Lux light for 15 min. The enzyme solution was replaced with phosphate buffer in both the positive and negative controls; the negative control was also stored in the dark. Then, a UV-2450 spectrophotometer (Shimadzu, Tokyo, Japan) was used to measure the absorbance at 560 nm. The positive control was set as the maximum, and the negative control was the blank control. The 50% inhibition of the photochemical reduction of tetrazolium blue per gram of plant tissue per minute was taken as a unit of SOD activity.

The flavonoid content was determined according to the method of Heimler et al. [37]. Leaves (0.05 g) were extracted with 1.5 mL of 95% methanol at 4 °C for 24 h. Then, 0.15 mL of sample extract, 1.85 mL of deionized water, 0.2 mL of 5% NaNO_2_, 0.3 mL of 10% AlCl_3_ and 1 mL of 1 M NaOH were added to a 5 mL centrifuge tube and mixed well. A UV-2450 spectrophotometer (Shimadzu, Tokyo, Japan) was used to determine the absorbance at 510 nm. Different concentrations of catechin were used to generate a standard curve to calculate the concentration.

The content of total phenol was determined according to the method of Ainsworth and Gillespie [38]. Leaves (0.05 g) were extracted with 1.5 mL of 95% methanol at 4 °C for 24 h. Then, 0.2 mL of sample extract, 0.8 mL of deionized water, 1 mL of 10% Folin and 2 mL of 0.7 M Na_2_CO_3_ were added to a 5 mL centrifuge tube and mixed. A UV-2450 spectrophotometer (Shimadzu, Tokyo, Japan) was used to determine the absorbance at 765 nm. Different concentrations of gallic acid were used to generate a standard curve to calculate the concentration.

The content of malondialdehyde (MDA) was determined according to the method of Sun et al. [20]. First, 0.2 g of sample was weighed into a mortar, then2 mL of 10% trichloroacetic acid was added for grinding; the sample was then centrifuged at 4 °C and 4000× *g* for 15 min. Next, 1 mL of supernatant was collected and mixed with an equal volume of 0.67% 2-thiobarbituric acid, and then the sample was boiled for 20 min. After cooling, a UV-2450 spectrophotometer (Shimadzu, Tokyo, Japan) was used to measure the absorbance at 600, 532 and 450 nm.

### 4.5. Determination of Net Photosynthetic Rate and Fluorescence Parameters

The net photosynthetic rate (Pn) was determined according to Zhang et al. [34]. The Pn was measured by an LI-6800 portable photosynthesis measurement system (LI-COR, USA). The light intensity of the detection chamber was set to 900 μmol m^−2^ s^−1^, the ratio of red to blue light was 9:1, and the concentration of CO_2_ was 400 μmol m^−2^ s^−1^. The humidity was 65% and the temperature was 28 °C. The Pn was recorded after the value was relatively stable.

A Chl fluorescence imaging system (Technologica, UK) was used to measure Chl fluorescence. The leaves were placed in the dark for 30 min. The minimum fluorescence (F_o_) and the maximum fluorescence (F_m_) of the dark-adapted leaves were measured using a 6100 μmol m^−2^ s^−1^ saturating pulse. The maximum photochemical efficiency (F_v_/F_m_) of photosystem II (PSII) was calculated as F_v_/F_m_ = (F_m_ − F_o_)/F_m_). The actual fluorescence (F′) and the maximum fluorescence (F_m_′) of the leaves exposed to light (PPFD = 900 μmol m^−2^ s^−1^) for five min were measured. The actual photochemical efficiency (Yield) was calculated as: Yield = (F_m_′ − F′)/F_m_′. Electron transport rate (ETR) was calculated as ETR = Yield × PPFD × 0.85 × 0.5, where the coefficient 0.85 was the leaf absorptance and the coefficient 0.5 indicated that the absorbed PPFD was equally allocated between PSI and PSII. Nonphotochemical quenching (NPQ) was calculated as NPQ = (F_m_/F_m_′) − 1 [39].

### 4.6. RNA-Seq Materials

After eight d of drought treatment, the third leaves of *S. calendulacea*, *S. trilobata* and their hybrid in the control group (normal irrigation) and drought treatment group were selected as the research materials. After cutting, the leaves were quickly put into the labeled sampling bag and stored in liquid nitrogen for subsequent total RNA extraction, with three biological replicates in each group. 

### 4.7. Total RNA Extraction, Library Construction and Transcriptome Sequencing

Total RNA was extracted from the leaves of the two parents and hybrids by TRIzol reagent (Invitrogen, MA, USA). The RNA quality of the samples was determined by ultraviolet spectrophotometry and agarose gel electrophoresis. The purity of the RNA was evaluated by the absorbance ratio of OD_260/280_ and OD_260/230_, the integrity of the RNA was confirmed by 1% agarose gel electrophoresis. The RNA was quantified by Qubit, and samples with poor quality were extracted again.

Using the Biomarker RNA library preparation kit, the library was constructed from the total RNA samples of the leaves of the two parents and hybrids. The quality of the constructed library was detected by an Agilent 2100 biological analyzer and an ABI Step One Plus Real-Time PCR system. After determining the quality detection, paired-end sequencing was performed on an Illumina HiSeq 2000 platform. Raw Illumina sequences and assembled sequences are available in the Gene Expression Omnibus (GEO) database of the National Center for Biotechnology Information (NCBI) (accession number: SUB9951500).

### 4.8. Assembly and Unigene Functional Annotation after Sequencing

To obtain high-quality data (clean reads), the original reads were filtered to delete low-quality sequences. Clean reads were assembled by Trinity and then clustered by CD-HIT. The remaining sequences are defined as unigenes.

To obtain their direction, function and path annotation, unigenes were searched in the nonrenewable protein (NR) database, Swiss-Prot protein database and Kyoto Encyclopedia of Genes and Genomes (KEGG) pathway database. Blast2GO software was used to annotate unigenes with Gene Ontology (GO) to describe the biological process, molecular function and cellular composition of these genes.

### 4.9. Real-Time PCR

Gene expression analysis was conducted according to the method published by Zhang et al. [34]. The genes’ relative expression levels were analyzed using the methods of Livak and Schmittgen [40]. The *GAPDH* gene was used as an internal reference, and for a list of PCR primers, see Supplementary Table S1.

### 4.10. Statistical Analysis

Statistical significance was determined by one-way analysis of variance (ANOVA) followed by Duncan’s *post hoc* test using SPSS Statistics 19.0 (IBM, Armonk, NY, USA). The significance was set as *p* < 0.05. SigmaPlot 12.5 (Systat Software Inc., Richmond, VA, USA) was used to conduct linear regression analysis and to plot the data.

## 5. Conclusions

In conclusion, under drought stress, *S. trilobata* can rapidly synthesize a large amount of ABA and reduce stomatal opening and water loss. Moreover, the antioxidant capacity of *S. trilobata* was the strongest, while that of *S. calendulacea* was the weakest. Among the three species, the drought tolerance of *S. trilobata* was the strongest and that of *S. calendulacea* was the weakest; the hybrid was between the values of the two parents. The results suggest that with the frequent occurrence of global warming and the continued intensifying of arid climates in the future, the alien invasive plant *S. trilobata* will have a greater competitive advantage in survival, the living space of local species *S. calendulacea* may be further compressed, and the invasion of *S. trilobata* will be further intensified.

## Figures and Tables

**Table 1:** DEGs under normal irrigation and drought treatment of three species.

Species	Up-Regulated	Down-Regulated	Total
*S*. *calendulacea*	307	240	547
*S*. *trilobata*	781	1151	1932
Hybrid	882	293	1115

## Supplementary Materials

The following are available online at www.mdpi.com/1422-0067/222/1/1288/s1.
