# Supplementary material for: Responses of Sphagneticola trilobata, Sphagneticola calendulacea and Their Hybrid to Drought Stress
Source: Int J Mol Sci. 2021 Oct 19;22(20):11288. doi: 10.3390/ijms222011288 (PMC8538449; doi:10.3390/ijms222011288)
Supplement: Supplementary file 1 [file ijms-22-11288-s001.zip › ijms-1392518-supplementary.pdf]

Supplementary Figures

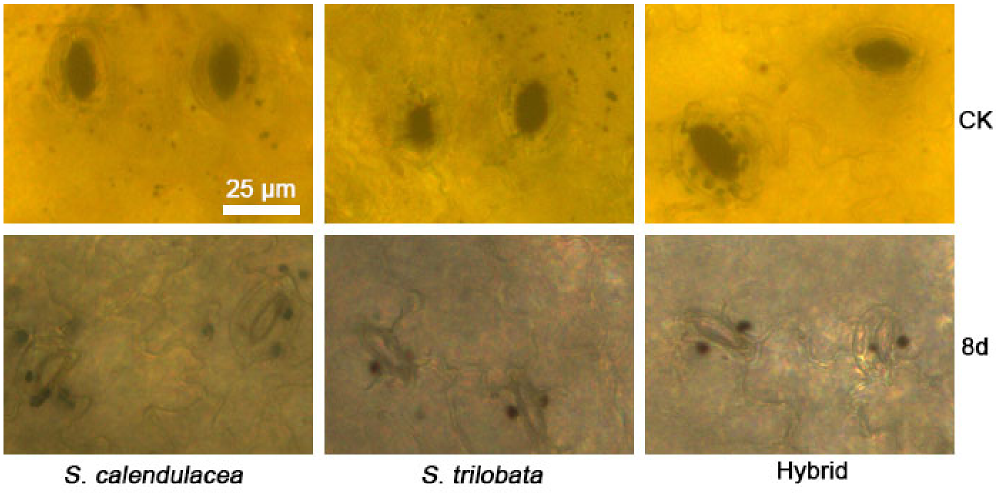

Supplementary Figure S1. After 8 days of normal irrigation (CK) and drought stress treatment (8d), potassium ion localization in leaves of *S. calendulacea*, *S. trilobata* and their hybrid.

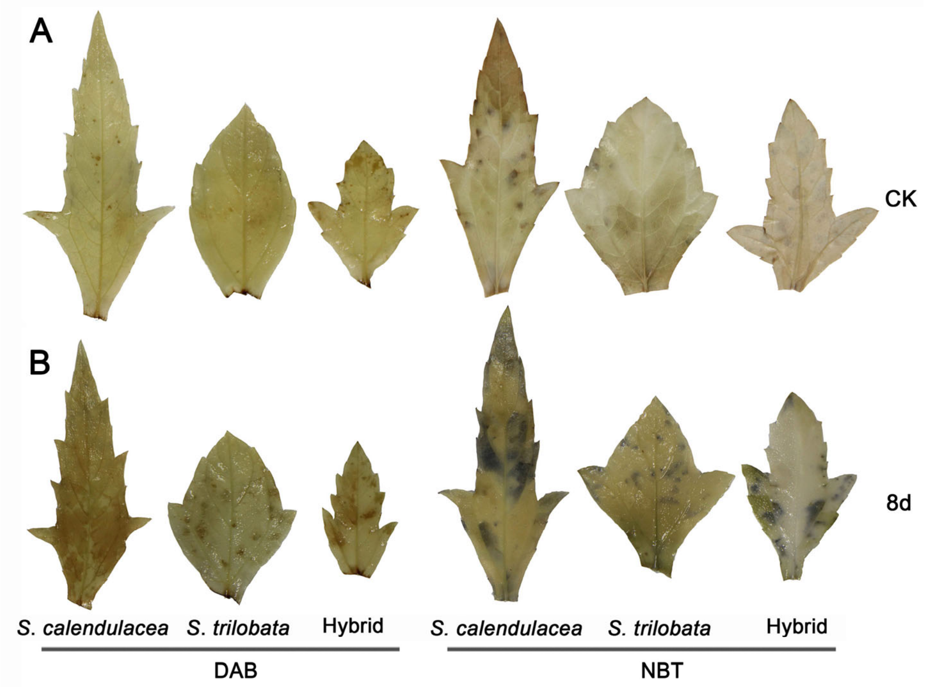

Supplementary Figure S2. Hydrogen peroxide (3,3'-diaminobenzidine (DAB) staining, A) and superoxide anion (nitroblue tetrazolium (NBT) staining, B) localization in leaves of *S. calendulacea*, *S. trilobata* and their hybrid after 8 days of normal irrigation (CK) and drought stress treatment (8d).

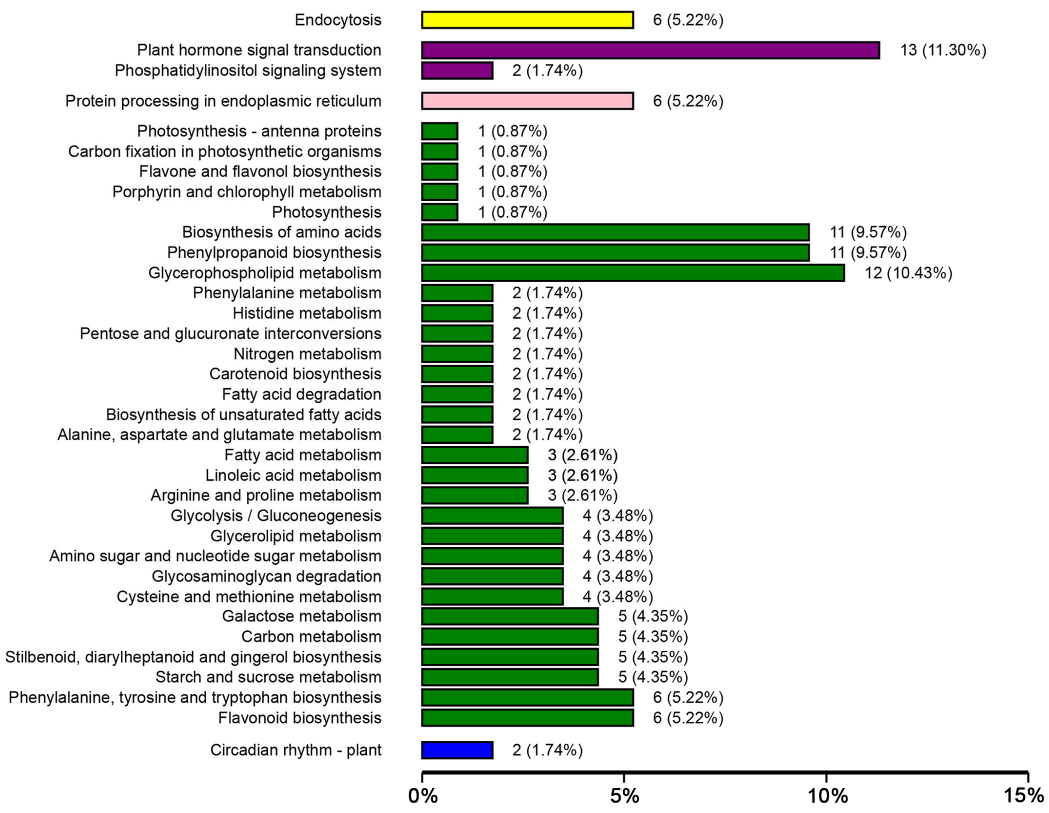

Supplementary Figure S3. KEGG classification of differentially expressed genes in *S. calendulacea* under normal irrigation (control) and drought treatment (treatment).

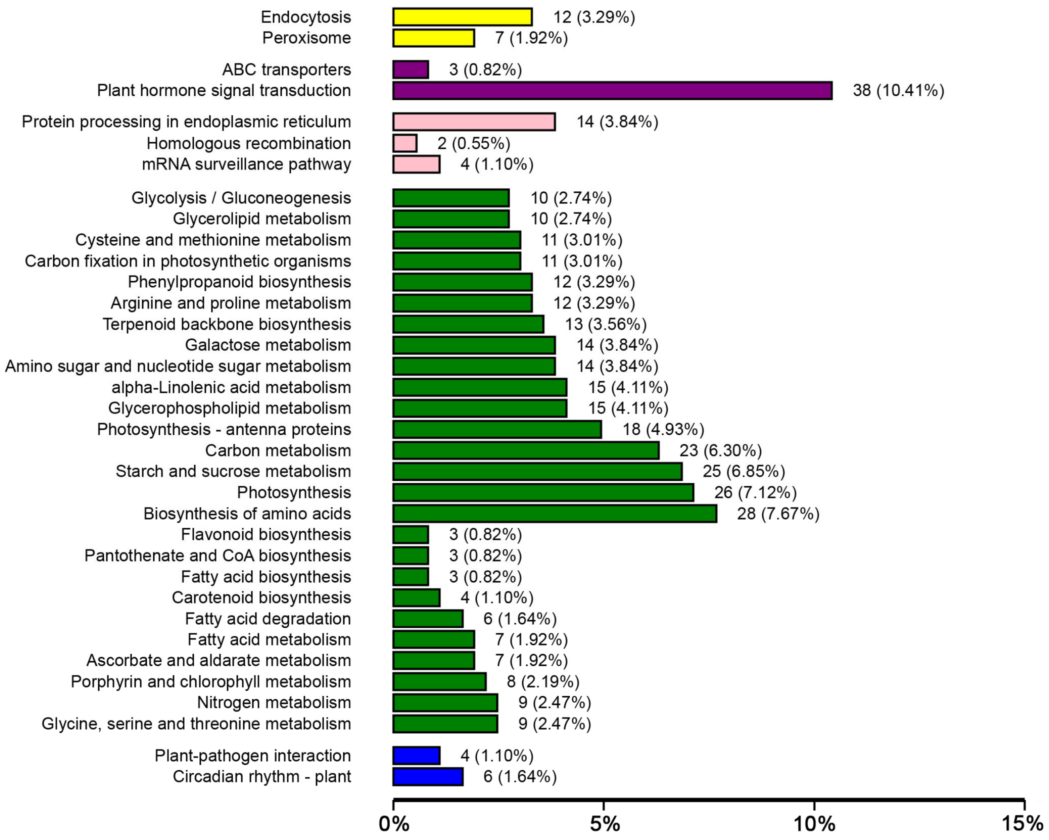

Supplementary Figure S4. KEGG classification of differentially expressed genes in *S. trilobata* under normal irrigation (control) and drought treatment (treatment).

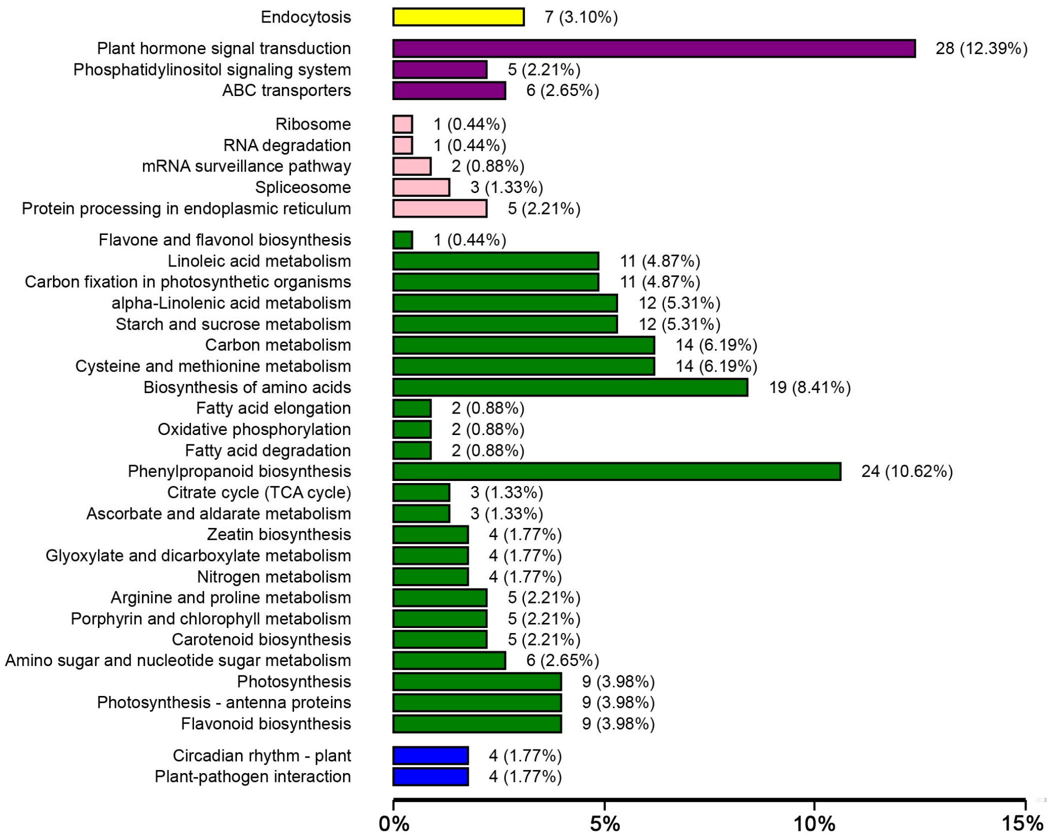

Supplementary Figure S5. KEGG classification of differentially expressed genes in hybrid under normal irrigation (control) and drought treatment (treatment).

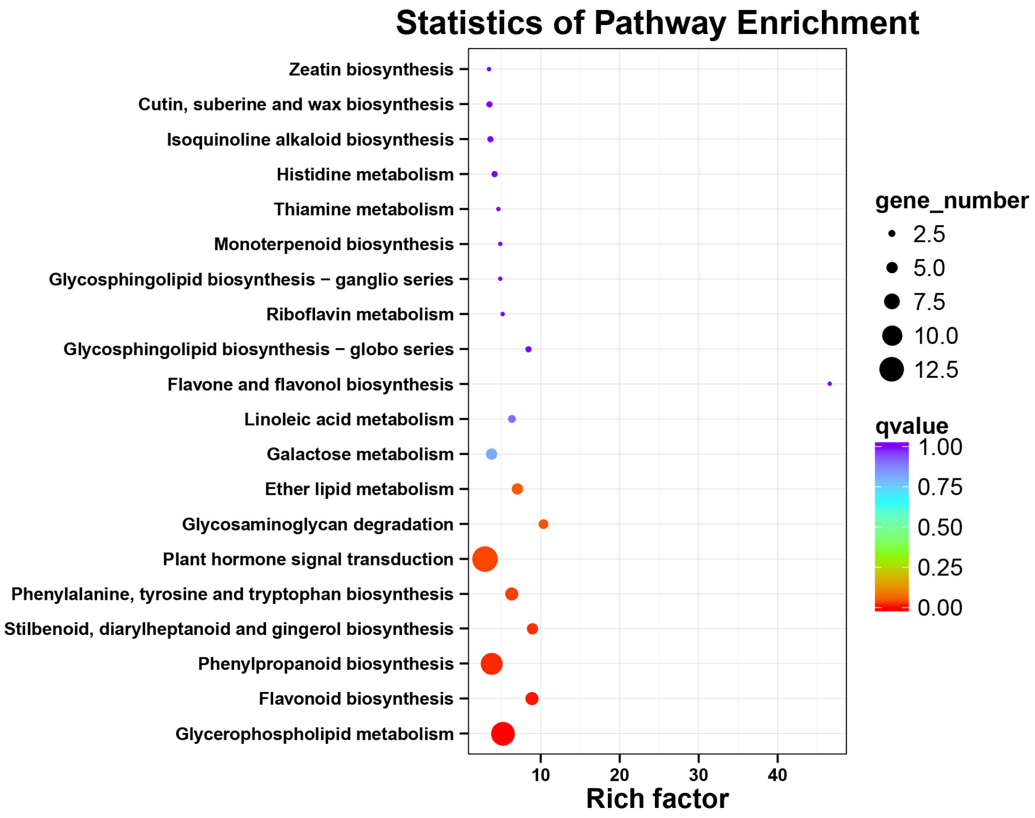

Supplementary Figure S6. Scatter plot of KEGG pathway enrichment of differentially expressed genes in *S. calendulacea* under normal irrigation (control) and drought treatment (treatment).

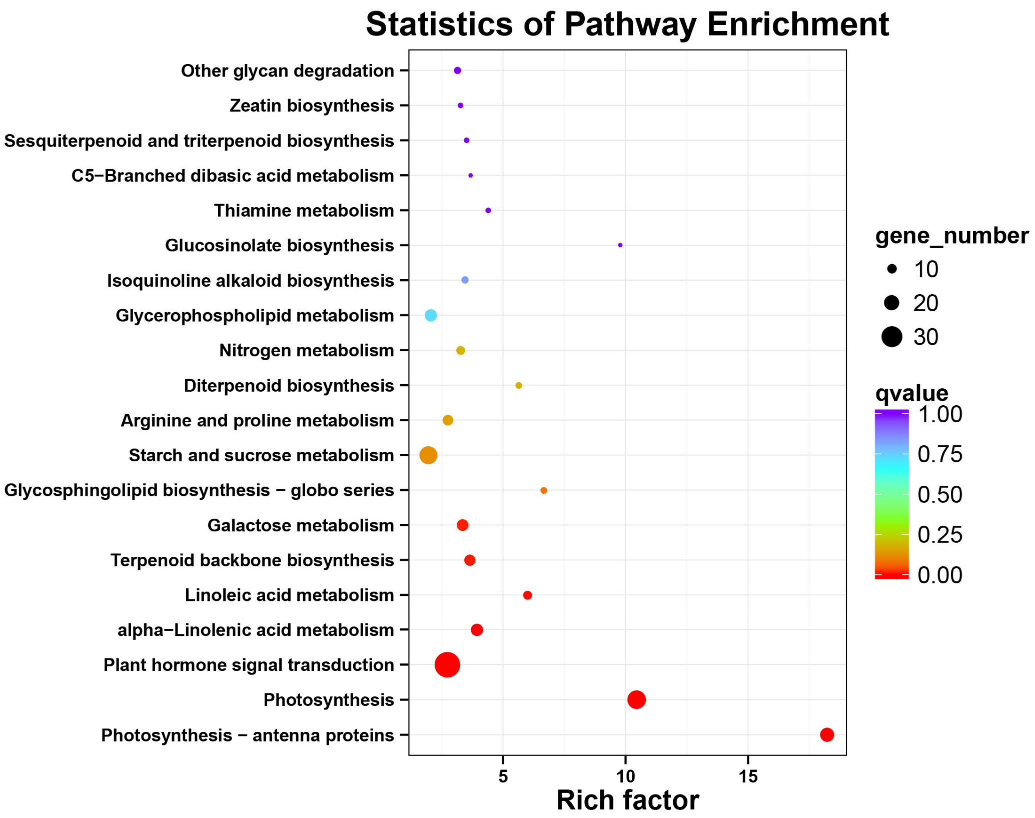

Supplementary Figure S7. Scatter plot of KEGG pathway enrichment of differentially expressed genes in *S. trilobata* under normal irrigation (control) and drought treatment (treatment).

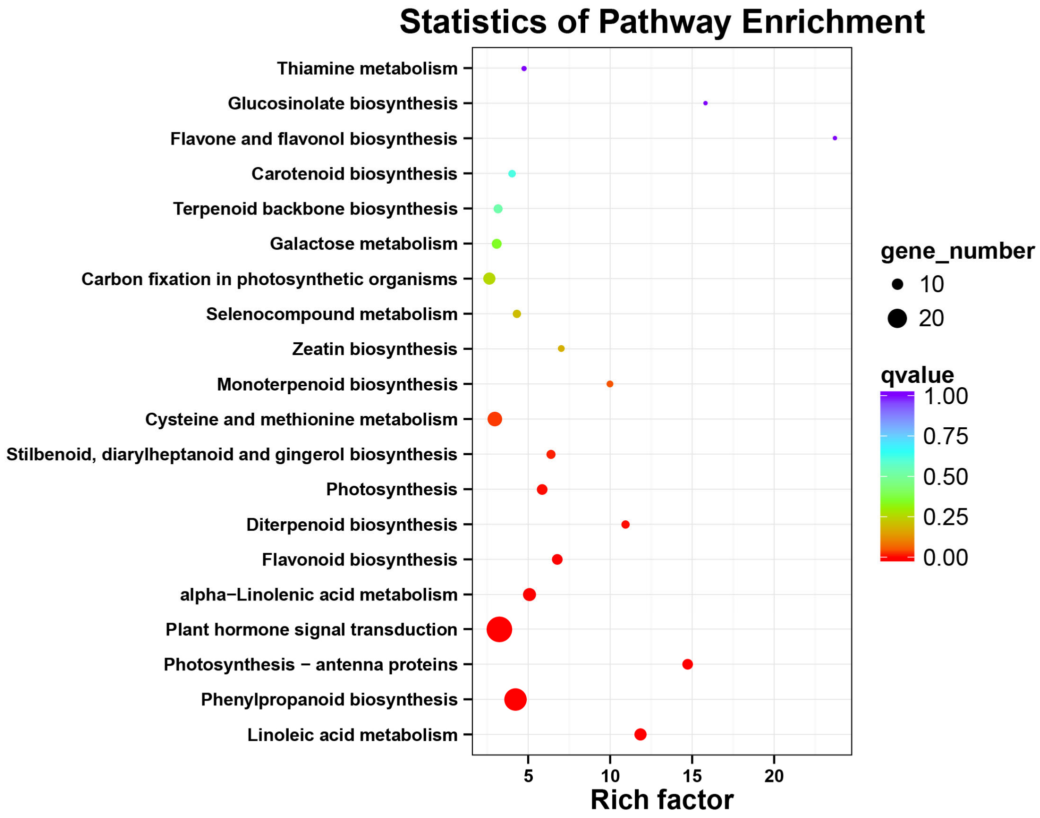

Supplementary Figure S8. Scatter plot of KEGG pathway enrichment of differentially expressed genes in hybrid under normal irrigation (control) and drought treatment (treatment).

Supplementary Table

Supplementary Table S1. Primers used for Real-Time PCR analysis

Gene ID (Gene name)	Primer sequence (5′-3′)	Gene description
c185702.graph_c0 (*GAPDH*)	F: GGCTCGACTCGGCATATTCT<br>R: CGGCTGCCTTTGGTCTATGT	Internal reference
c181991.graph_c0 (*PGK*)	F: ACCGATAAACCTTGCGCCTT<br>R: GATTACCTTGATGGGGCGGT	Calvin cycle
c173064.graph_c3 (*RubL*)	F: CGCCTCACGGTATCCAAGTT<br>R: TCTTCGCAATTACCCGCAGT	Calvin cycle
c189563.graph_c0 (*P5CS*)	F: GGGCAAGTGGTAGAAGGTGA<br>R: ACTCTACTTGACGAAACAAACC	Proline synthesis
c180187.graph_c1 (*Fructofuranosidase*)	F: CAAACTTTAAGGCTGCCCCC<br>R: TTGGAAGATGGGTGGAGCAA	Sugar synthesis
c191254.graph_c0 (*Glucosidase*)	F: CGCGACCGCTATTCTTCACA<br>R: CATCAACAGAGACTGCCCCC	Sugar synthesis
c176762.graph_c0 (*NCED*)	F: GGCATTGGACGCGATTGAAG<br>R: TCGAACAACGGGTTAGCTCC	ABA synthesis
c188273.graph_c1 (*ABA1*)	F: TAGCCCAACAACACTTCACTCA<br>R: AGCCACCAACACCCTTATGT	ABA synthesis
c182856.graph_c1 (*ABF*)	F: GCGAGATGACCCTTGAGGAG<br>R: TCCCAGTTCCACCCTGTTGA	ABA signal transduction
c188492.graph_c0 (*PsaA*)	F: GGGGCAAGTGTTCGGATCTATTA<br>R: AAAAACATGAAGCAGCGCCAC	Photosynthesis
c168832.graph_c2 (*PsbD*)	F: ACTGGAAATGAGAGTATTTATCCCG<br>R: AGCTATCAGATCGGGCTTGATG	Photosynthesis
c178623.graph_c0 (*Lhca2*)	F: TGTTTGCTCACCTTGCTGAC<br>R: CTTGGCCTACAATCAATCCTCCT	Chlorophyll synthesis
c183538.graph_c0 (*Lhcb1*)	F: AGCAACAACGCTTGGGCTTA<br>R: CTTGTTGATAAGACACCCGCA	Chlorophyll synthesis
c117063.graph_c0 (*CAT*)	F: CGCAGAGTTGCAGAAGGTCT<br>R: ACAATAGGCACCGCCATAGG	CAT synthesis
c175474.graph_c0 (*SOD*)	F: GAACCCAAGATTCCGGTTGC<br>R: TTGGAGGAAAACTGACTGCAT	SOD synthesis
c176172.graph_c0 (*POD*)	F: ATGCGGGTACATTTAGAGGGT<br>R: ACACAGGTAACCATCAACCATA	POD synthesis

Supplementary Methods

Potassium ion localization

The localization of potassium ion was according to the method described by Zhang et al. [1] with slightly changed. Prepare the first solution: dissolve 4.6 g of $NaNO_2$ in 10 ml of deionized water, and then added 3.2 ml of 6 M acetic acid. Then 0.8 g of $Co(NO_3)_2$ and 0.5 g of $Pb(NO_3)_2$ were mixed and added to the first solution. Store at 4 °C for 12 h after mixing, and save for use after filtering and avoid light at 4 °C. After cleaning, the leaves were put into the above solution containing 15%, vacuumized for 10 min and soaked in dark for 5 min. Then wash the leaves with 50% ethanol for 3 times, vacuum the leaves with $(NH_4)_2S$ containing 5% for 5 min, soak them in dark for 5 min, wash the leaves with 50% ethanol for 3 times and decolorize them in 80% acetone. Under the light microscope, the brown black crystal in the leaves was cos precipitation.

Tissue localization of hydrogen peroxide and superoxide anion

3,3'-diaminobenzidine (DAB) and nitroblue tetrazolium (NBT) were used to determine hydrogen peroxide and superoxide anion in leaves, respectively [2]. The leaves were soaked in 0.5 mg ml^{-1} DAB solution (dissolved in 0.05 M phosphate buffer with pH 7.0), vacuumized and soaked in dark for 4 h, and then decolorized with 80% acetone. Hydrogen peroxide and DAB form brown matter in leaves. The leaves were soaked in 1 mg ml^{-1} NBT solution (dissolved in 0.05 M phosphate buffer solution with pH 6.4), vacuumized and soaked in dark for 4 h, and then decolorized with 80% acetone. The superoxide anion in leaves and NBT form blue substance in leaves.

Supplementary References

1. Zhang, L.; Wang, Q.; Li, S.; Dong, H.; Yao, Y. *In situ* detection technology of potassium in submicroscopic structures of plants. *Plant Physiol. J.* **2015**, *51*, 1524–1528.
2. Liu, Y.; Ren, D.; Pike, S.; Pallardy, S.; Gassmann,W.; Zhang, S. Chloroplast-generated reactive oxygen species are involved in hypersensitive response-like cell death mediated by a mitogen-activated protein kinase cascade. *Plant J.* **2007**, *51*, 941–954.
